# Role of Personality and Positive and Negative Affects in Coping Strategies of Nurses: A Cross-Sectional Study

**DOI:** 10.3389/fpsyg.2021.682153

**Published:** 2021-09-03

**Authors:** África Martos Martínez, María del Mar Molero Jurado, María del Carmen Pérez-Fuentes, Ana Belén Barragán Martín, María del Mar Simón Márquez, José Jesús Gázquez Linares

**Affiliations:** ^1^Department of Psychology, Faculty of Psychology, University of Almería, Almería, Spain; ^2^Department of Psychology, Universidad Politécnica y Artística del Paraguay, Asunción, Paraguay; ^3^Department of Psychology, Universidad Autónoma de Chile, Providencia, Chile

**Keywords:** personality, affect, coping strategies, nurses, descriptive of survey study

## Abstract

Nurses are exposed to many highly stressful events. Individual variables, such as personality and affective state, have been related to vulnerability to maladaptive coping. Therefore, the objectives of this study were to analyze the relationships between the personality, positive and negative affects, and coping strategies of nurses and to establish the mediating role of affective state in the relationship between personality and coping. The sample was made up of 1,268 Spanish nurses aged 22–63 years who completed the Coping Strategies Inventory, the Positive and Negative Affect Schedule, and the 10-item Big Five Inventory. Descriptive analyses, correlations, and mediation models were estimated. The results showed relationships between the Big Five personality factors, positive and negative affects, and coping strategies. Negative affect was confirmed as a mediator between personality and less adaptive strategies and positive affect was confirmed on positive strategies. This study emphasizes the need to develop actions directed at teaching nurses adequate problem-solving strategies and training them in the ability to assign a different emotional value to complex situations.

## Introduction

In spite of the importance of wellbeing of healthcare professionals in ensuring quality care and patient safety (Hall et al., 2016), this group is habitually exposed to highly stressful situations (Pérez-Fuentes et al., 2019a; Tirado et al., 2019; Wijn and van der Doef, 2020). This generates special vulnerability in nurses to develop burnout (Molero et al., 2018a,b), depression, and negative moods (Schürmann and Magraf, 2018; Veiga et al., 2019).

### Coping Strategies in Nursing

Coping refers to cognitive and behavioral responses of an individual in confronting and managing stressful situations (Folkman, 2013). Not all forms of coping are considered beneficial. Adopting strategies, such as self-blame, or problem avoidance as a way of struggling with stressors lead to maladaptive results (Mark and Smith, 2012; Morales, 2020). Thus, coping responses may be divided into positive and negative. Positive coping refers to direct rational problem-solving, while negative coping refers to avoiding, withdrawing, or denying the problem (Jia et al., 2004). The professionals who use dysfunctional coping strategies show more emotional exhaustion and anxiety, which in turn can lead to severe consequences, such as substance abuse, negligence, or changing profession (Bamonti et al., 2019). Similarly, Voskou et al. (2020) showed that nurses who use strategies related to imagining and wishing for alternative scenarios or for escape and problem avoidance have a worse quality of life due to deterioration of their physical and mental health. In this regard, in addition to poor general health, passive coping has also been related to low control and scant job support (Schreuder et al., 2012).

While strategies, such as self-criticism, social withdrawal, and problem avoidance, generate a risk for moral disconnection in the nursing practice, the development of successful strategies enables nurses to advance in their professional labor (Forozeiya et al., 2019). Positive coping skills, such as the use of problem-focused strategies, are related to high standards in resolving complicated situations, better capacity for recovery and resilience, and a healthier work environment (Schreuder et al., 2012; Teismann et al., 2018; Yu et al., 2019). Likewise, employing a positive focus in solving problems and seeking social and emotional support are related to better physical and psychological health of nurses (Voskou et al., 2020).

### Personality and Affect in Nursing

Along with the strategies deployed for facing stressful situations, there are other individual characteristics that affect the job performance and quality of life, such as personality and affective state, of a professional (Barr, 2018). Personality particularly influences a variety of important results in different areas of life of a person, among which is work (Serrano-Fernández et al., 2019). In this line, following the Big Five model, three personality profiles were found in nurses: one group with high scores in extraversion traits, agreeableness, openness to experience and conscientiousness, and low in neuroticism; a second group with high scores in conscientiousness, openness to experience, and neuroticism, but low in agreeableness and extraversion; and a third group with very high scores in neuroticism and low in the rest of the variables. The two last profiles, but especially the one marked by high neuroticism, have been associated with stronger affects by stressful work situations, resulting in a higher prevalence of burnout (Pérez-Fuentes et al., 2019b).

Following Davis and Panksepp (2011), personality and affective states are closely related, and to know the human personality, affective forces and emotional impulses must be understood. With regard to personality and its link with affect, neuroticism has been associated with negative affective state, while conscientiousness, agreeableness, openness, and, especially, extraversion have been linked with positive affect (Hengartner et al., 2017).

### Coping Strategies and Personality: Mediation of Positive and Negative Affects

According to Sahler and Carr (2009), personality characteristics, along with attributional style, form the coping style of an individual. Specifically, the relationship between personality and certain coping styles is due to the personality being able to moderate physiological excitation caused by stress, altering evaluation of the situation, and, therefore, the choice and effectiveness of coping (Wiebe et al., 2018). It, therefore, seems evident that personality traits influence coping (Anisman et al., 2018). So, for example, highly conscientious people tend to use proactive, problem-focused strategies (Straud et al., 2015), and at the same time, they usually feel less anxious and more positive affect than individuals with lower scores in this trait (Korotkov, 2008). Moreover, professionals with a personality marked by neuroticism may try to solve everyday problems with emotional strategies because of their high emotional reactiveness, instead of choosing more effective strategies, such as those focused on the problem (Fornés-Vives et al., 2019). Individuals with high levels of this trait choose more or less regulating responses depending on the affective burden of the situation (Augustine and Larsen, 2011).

Interaction between coping strategies and affect must therefore be considered to understand the regulation dynamics in addition to individual personality differences (Pavani et al., 2017; De la Barrera et al., 2019). It seems that people who feel negative affect at a certain time focus on negative stimuli and restrict their behavior to meditation on past or future problems to overestimate and then avoid the risk, which, in turn, can generate new negative affective experiences (Teismann et al., 2019). Along this line, a negative coping style avoiding the problem has been related to negative affect, while a problem-focused coping is related to positive affect (Dunkley et al., 2017). But further, within this relationship between affect and regulation strategies, personality has a very relevant role. Extraversion and neuroticism are the specific personality traits most involved. Extraversion provokes an initially positive affect in a stressful situation, which, in turn, leads to adaptive coping, while those more neurotic tend to experience negative affect more intensely, which involves the use of rumination and avoidance strategies (Pavani et al., 2017).

As research should concentrate on optimizing the performance and wellbeing of nurses (Dykstra et al., 2016), and nurses must frequently face stressful situations, coping is an important health process which must be included in this collective study (Kellog et al., 2018). Even though personality is relatively stable, coping strategies may be modeled (Wechler et al., 2018). The acquisition and development of effective coping strategies could therefore alleviate anxiety and increase the wellbeing of healthcare professionals (Huang et al., 2018; Molero et al., 2019). Thus, the objective of this study was to analyze the relationships between Big Five personality characteristics, affect (positive and negative), and coping strategies in a sample of nurses and to find out the mediator role of affect in the relationship between personality and coping strategies. The main hypothesis was that positive affect mediates the relationship between personality and more adaptive coping strategies, while, on the contrary, the negative affect mediates the relationship between personality and dysfunctional coping strategies.

## Method

### Participants

The original sample was made up of a total of 1,383 nurses in Andalusia (Spain). Those who were unemployed at the time of data collection were excluded (−68). Then, random or incongruent answers detected by control questions (CQ) were discarded. After filtering, the final sample was therefore comprised of 1,268 nurses. The mean participant age was 32.02 (*SD* = 6.91) in a range of 22–63 years. The sex distribution of the sample was 85.3% (*n* = 1,081) women and 14.7% (*n* = 187) men, with a mean age of 32.24 (*SD* = 6.68) and 32.79 (*SD* = 6.27), respectively.

### Instruments

The sociodemographic characteristics were evaluated with an *ad hoc* questionnaire including items on participant gender, marital status, and age.

How stressful events are coped with was evaluated using the *Inventario de Estrategias de Afrontamiento* [Coping Strategies Inventory] (CSI; Cano-García et al., 2007). This instrument consists of 40 items with a five-point Likert-type response scale. It has eight subscales grouped in two secondary scales: adequate management (e.g., *I fought to solve the problem*), well-focused on the problem (Problem-Solving and Cognitive Restructuring subscales) and well-concentrated on the emotion (Social Support and Emotion Expression subscales); and inadequate management (e.g., *I blamed myself*), problem focused (Problem Avoidance and Wishful Thinking) and emotion focused (Social Withdrawal and Self-Criticism subscales). The reliability indices were: *ω* = 0.883, the greatest lower bound (GLB) = 0.869 in problem-solving; *ω* = 0.866, GLB = 0.866 in self-criticism; *ω* = 0.823, GLB = 0.843 in emotion expression; *ω* = 0.869, GLB = 0.881 for wishful thinking; *ω* = 0.904, GLB = 0.930 for social support; *ω* = 0.818, GLB = 0.828 for the cognitive restructuring subscale; *ω* = 0.749, GLB = 0.787 on problem avoidance; and *ω* = 0.784, GLB = 0.824 for social withdrawal.

The Spanish version (Joiner et al., 1997) of the *Positive and Negative Affect Schedule* (PANAS Scale; Watson et al., 1988) was used for evaluating the positive and negative affects. This test is comprised of 20 items organized into two groups, 10 on positive affect (e.g., *Interested in things*) and 10 on negative affect (e.g., *Aggressive*). The answers are rated on a Likert-type scale in a range of 1–5 points. Reliability was *ω* = 0.753, GLB = 0.798 for positive affect and *ω* = 0.736, GLB = 0.822 for negative affect.

The 10-item Big Five Inventory (BFI-10; Rammstedt and John, 2007) was used to evaluate the personality traits. This provides information on the Big Five personality factors (extraversion, conscientiousness, agreeableness, neuroticism, and openness) (e.g., *I look like someone who is reserved*). Findings support the factorial validity, construct validity, and criterion validity of the BFI-10 (Rammstedt and John, 2007; Rammstedt et al., 2014).

### Procedure

The sample for this cross-sectional study was found by snowball sampling, by publishing it on social networks and texting. A computer-aided web interviewing (CAWI) survey was used to collect the data.

Before data were collected, the participants were assured that data processing in the study would comply with the applicable standards of data security, confidentiality, and ethics. The study was approved by the University of Almería Bioethics Committee, Spain (Ref: UALBIO2017/011). The questionnaire was administered in 2017 on a Web platform that enabled the participants to fill it in online. A series of control questions were inserted to control for random or incongruent answers, which were then eliminated from the study.

### Data Analysis

The study was quantitative, observational, and cross-sectional. First, Pearson's correlation analyses were performed. A Bayesian analysis was done of the pairs in which significance was from 0.01 to 0.05 for a more accurate statistic in the comparison of hypotheses. This evaluates the predictive adequacy of the null hypothesis, H_0_: *ρ* = 0, which stipulates that the correlation is absent, and compares it with the predictive adequacy of an alternative hypothesis H_1_, which stipulates that the correlation exists (Wagenmakers et al., 2016).

Mediation models were proposed based on the results found from the correlation matrices. Two mediation analyses were performed with multiple predictors, one mediator and four result variables, using JASP version 0.11.1 (JASP Team, 2019), based on the lavaan software (Rosseel, 2012). To test whether there was an indirect effect, the bias-corrected bootstrap confidence interval was applied (Biesanz et al., 2010). To examine the reliability of the instruments used for collecting data, the McDonald's Omega coefficient was estimated, following the proposal and instructions of McDonald (1999); Ventura-León and Caycho (2017). The GLB was also estimated.

## Results

### Preliminary Analyses

The mean scores and standard deviations of each of the study variables were as follows: Positive affect (*M* = 2.99, *SD* = 0.43), negative affect (*M* = 2.09, *SD* = 0.39), extraversion (*M* = 3.50, *SD* = 0.80), agreeableness (*M* = 4.01, *SD* = 0.58), conscientiousness (*M* = 3.87, *SD* = 0.67), neuroticism (*M* = 2.67, *SD* = 0.81), openness to experience (*M* = 3.51, *SD* = 0.74), problem solving (*M* = 13.59, *SD* = 3.96), self-criticism (*M* = 6.05, *SD* = 4.16), emotion expression (*M* = 10.31, *SD* = 3.83), wishful thinking (*M* = 10.67, *SD* = 4.73), social support (*M* = 13.27, *SD* = 4.40), cognitive restructuring (*M* = 11.75, *SD* = 3.91), problem avoidance (*M* = 6.98, *SD* = 3.74), and social withdrawal (*M* = 4.76, *SD* = 3.49).

Table 1 shows the pairwise correlation matrix for the affect and personality factors. Positive affect correlated positively with extraversion, agreeableness, conscientiousness, and openness to experience and had a negative relationship with neuroticism. Negative affect correlated positively with neuroticism and negatively with the rest of personality factors.

**Table 1:** Affect and personality—Pearson's pairwise correlations.

**Affect**	**Personality**	**Pearson's *r***	***p***	**Below 95% CI**	**Above 95% CI**
Positive	Extraversion	0.126	<0.001	0.071	0.179
	Agreeableness	0.106	<0.001	0.051	0.160
	Conscientiousness	0.202	<0.001	0.149	0.255
	Neuroticism	−0.146	<0.001	−0.200	−0.092
	Openness	0.177	<0.001	0.123	0.230
Negative	Extraversion	−0.072	0.011	−0.126	−0.017
	Agreeableness	−0.108	<0.001	−0.162	−0.053
	Conscientiousness	−0.143	<0.001	−0.197	−0.089
	Neuroticism	0.256	<0.001	0.203	0.306
	Openness	−0.060	0.032	−0.115	−0.005

The Bayes factor for negative affect-extroversion was (BF_−0_ = 1.802, 95% CI = −0.126, −0.019), which indicates that the data are 1.8 times more likely under the alternative hypothesis that expresses the existence of a negative correlation than under the null hypothesis. In line with Jeffreys (1961), a Bayes factor from 1 to 3 is considered weak or inconclusive evidence, a Bayes factor from 3 to 10 is considered moderate evidence, and a Bayes factor above 10 is considered strong evidence.

In the negative affect-openness to experience pair, the Bayes factor was (BF_−0_ = 0.684, 95% CI = −0.115, −0.011), which shows that the data are 1.46 times more likely under the null hypothesis than under the alternative hypothesis.

Table 2 presents the pairwise correlations between the affects and coping strategies. In the first place, problem-solving coping strategies and seeking social support were positively related with positive affect, while the other strategies based on self-criticism, wishful thinking, and social withdrawal showed positive correlations with negative affect.

**Table 2:** Affect, personality, and coping strategies—Pearson's pairwise correlations.

	**Coping strategies**	**Pearson's *r***	***p***	**Below 95% CI**	**Above 95% CI**
**Affect**					
Positive	- Problem-solving	0.300	<0.001	0.249	0.349
	- Self-criticism	0.034	0.221	−0.021	0.089
	- Emotion expression	0.211	<0.001	0.158	0.263
	- Wishful thinking	0.052	0.065	−0.003	0.107
	- Social support	0.254	<0.001	0.202	0.305
	- Cognitive restructuring	0.274	<0.001	0.223	0.324
	- Problem avoidance	0.094	<0.001	0.039	0.148
	- Social withdrawal	−0.013	0.635	−0.068	0.042
Negative	- Problem-solving	−0.035	0.211	−0.090	0.020
	- Self-criticism	0.292	<0.001	0.240	0.341
	- Emotion expression	0.125	<0.001	0.071	0.179
	- Wishful thinking	0.111	<0.001	0.056	0.165
	- Social support	0.021	0.457	−0.034	0.076
	- Cognitive restructuring	0.077	0.006	0.022	0.131
	- Problem avoidance	0.163	<0.001	0.109	0.216
	- Social withdrawal	0.254	<0.001	0.202	0.305
**Personality**					
Extraversion	- Problem-solving	0.212	<0.001	0.159	0.264
	- Self-criticism	−0.165	<0.001	−0.218	−0.110
	- Emotion expression	0.215	<0.001	0.161	0.267
	- Wishful thinking	−0.035	0.218	−0.089	0.020
	- Social support	0.267	<0.001	0.215	0.318
	- Cognitive restructuring	0.140	<0.001	0.085	0.193
	- Problem avoidance	−0.028	0.321	−0.083	0.027
	- Social withdrawal	−0.257	<0.001	−0.308	−0.205
Agreeableness	- Problem-solving	0.262	<0.001	0.210	0.313
	- Self-criticism	−0.090	0.001	−0.144	−0.035
	- Emotion expression	0.165	<0.001	0.111	0.218
	- Wishful thinking	0.088	0.002	0.034	0.143
	- Social support	0.239	<0.001	0.186	0.290
	- Cognitive restructuring	0.173	<0.001	0.119	0.226
	- Problem avoidance	−0.060	0.034	−0.114	−0.005
	- Social withdrawal	−0.201	<0.001	−0.253	−0.148
Conscientiousness	- Problem-solving	0.343	<0.001	0.293	0.391
	- Self-criticism	−0.211	<0.001	−0.263	−0.158
	- Emotion expression	0.081	0.004	0.026	0.135
	- Wishful thinking	−0.010	0.709	−0.066	0.045
	- Social support	0.162	<0.001	0.108	0.215
	- Cognitive restructuring	0.172	<0.001	0.118	0.225
	- Problem avoidance	−0.074	0.008	−0.129	−0.019
	- Social withdrawal	−0.203	<0.001	−0.256	−0.150
Neuroticism	- Problem-solving	−0.244	<0.001	−0.295	−0.192
	- Self-criticism	0.279	<0.001	0.228	0.329
	- Emotion expression	−0.027	0.340	−0.082	0.028
	- Wishful thinking	0.189	<0.001	0.135	0.241
	- Social support	−0.129	<0.001	−0.183	−0.075
	- Cognitive restructuring	−0.152	<0.001	−0.205	−0.097
	- Problem avoidance	0.011	0.688	−0.044	0.066
	- Social withdrawal	0210	<0.001	0.156	0.262
Openness to experience	- Problem-solving	0.249	<0.001	0.197	0.300
	- Self-criticism	−0.114	<0.001	−0.168	−0.059
	- Emotion expression	0.101	<0.001	0.047	0.156
	- Wishful thinking	0.004	0.881	−0.051	0.059
	- Social support	0.168	<0.001	0.114	0.221
	- Cognitive restructuring	0.143	<0.001	0.088	0.196
	- Problem avoidance	−0.005	0.846	−0.060	0.050
	- Social withdrawal	−0.100	<0.001	−0.154	−0.045

Furthermore, other coping strategies were identified that were positively related with both affects: emotion expression, cognitive restructuring, and problem avoidance.

Regarding the pairwise correlations between personality factors and coping strategies, coping strategies based on problem-solving correlated positively with extraversion, agreeableness, conscientiousness, and openness to experience, while the correlation with neuroticism was negative.

The self-criticism strategy was positively related to neuroticism and negatively to the rest of the personality factors.

Emotion expression showed positive correlations with all the personality factors except neuroticism with which it was not correlated.

Coping directed at wishful thinking correlated positively with neuroticism and, to a lesser extent, with agreeableness. Seeking social support correlated positively with all the personality factors and negatively with neuroticism, as did the cognitive restructuring strategy.

In addition, problem-avoidance coping was negatively related with agreeableness and conscientiousness.

Finally, social withdrawal was a coping strategy associated positively with neuroticism and negatively with the four remaining personality factors.

### Mediation Models

**Figure 1:**
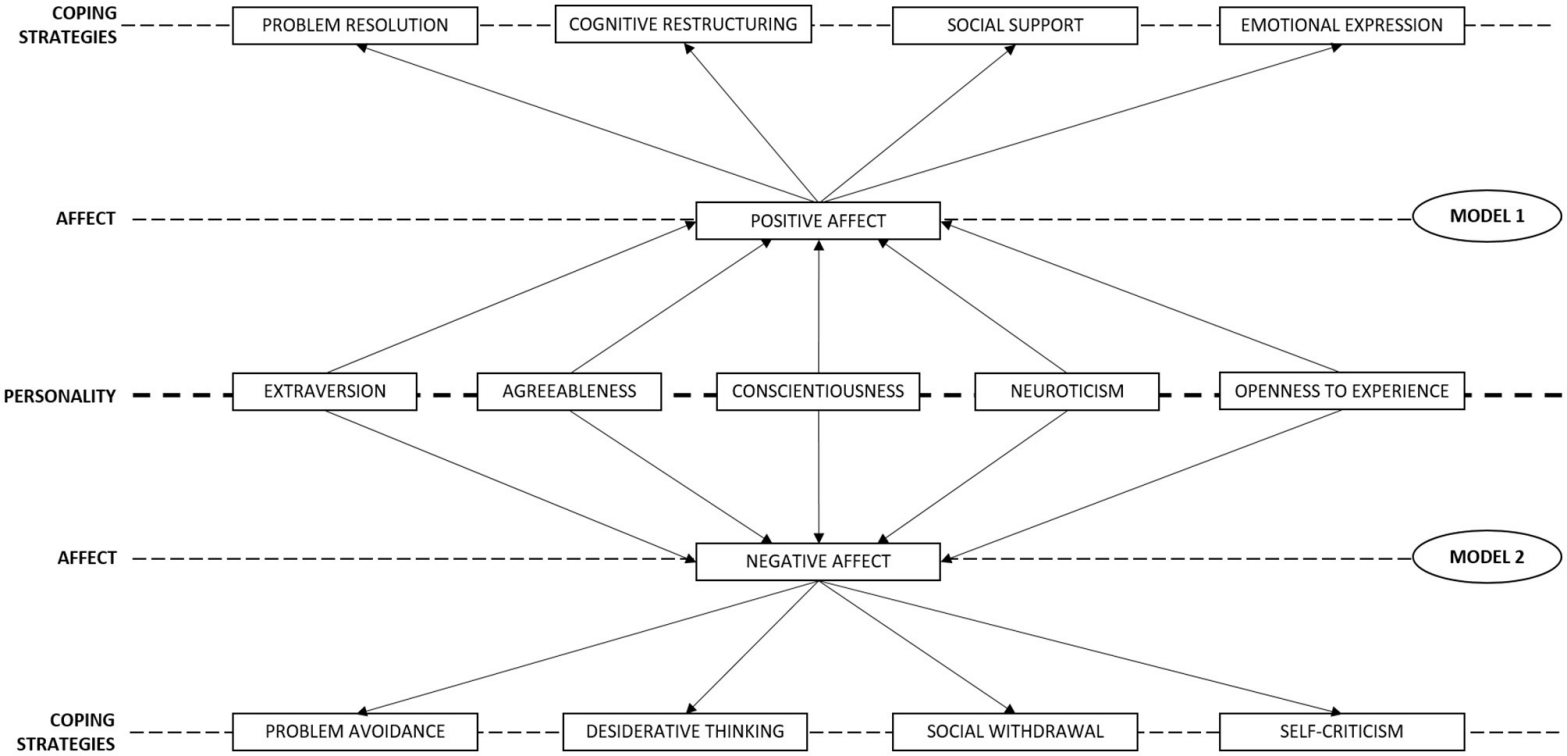
Hypothetical mediation models.

Two mediation models were proposed as follows (Figure 1):

Model 1, which proposes positive affect as a mediator in the relationship between the personality dimensions and positive coping strategies (considered as such based on the preliminary results of positive relationships of affect and the personality dimensions that could be beneficial, in this case, for professional practice).

Model 2 hypothesized the existence of a mediating effect of negative affect in the relationship established between the personality factors and the use of negative coping strategies.

As observed in Table 3, the extraversion and agreeableness factors showed direct positive effects on the “positive” coping strategies (problem-solving, cognitive restructuring, social support, and emotion expression). The conscientiousness factor had a direct positive effect on the problem-solving strategy and, to a lesser extent, on cognitive restructuring. On these two strategies, although negatively, a direct effect of neuroticism was observed, whereas the openness to experience factor exerted a direct positive effect on problem-solving and social support.

**Table 3:** Direct and indirect effects (Model 1).

	**Estimated**	**Standard error**	***z*-value**	***p***	**95% CI**	
					**Below**	**Above**
**Direct effects**						
EXT → PRS	0.107	0.032	3.361	<0.001	0.049	0.170
AGR → PRS	0.251	0.044	5.691	<0.001	0.169	0.343
CON → PRS	0.286	0.040	7.109	<0.001	0.202	0.372
NEU → PRS	−0.137	0.032	−4.315	<0.001	−0.196	−0.072
OPE → PRS	0.149	0.035	4.261	<0.001	0.084	0.226
EXT → COR	0.073	0.034	2.112	0.035	0.007	0.139
AGR → COR	0.182	0.047	3.834	<0.001	0.080	0.278
CON → COR	0.086	0.043	1.988	0.047	−2.043e−4	0.174
NEU → COR	−0.085	0.034	−2.499	0.012	−0.155	−0.014
OPE → COR	0.073	0.038	1.932	0.053	0.003	0.149
EXT → SOS	0.236	0.034	7.040	<0.001	0.169	0.306
AGR → SOS	0.271	0.046	5.849	<0.001	0.181	0.362
CON → SOS	0.037	0.042	0.866	0.387	−0.053	0.128
NEU → SOS	−0.030	0.033	−0.904	0.366	−0.096	0.037
OPE → SOS	0.103	0.037	2.814	0.005	0.025	0.182
EXT → EME	0.216	0.035	6.223	<0.001	0.145	0.288
AGR → EME	0.195	0.048	4.051	<0.001	0.101	0.287
CON → EME	−0.015	0.044	−0.348	0.728	−0.110	0.070
NEU → EME	0.064	0.034	1.864	0.062	−0.011	0.135
OPE → EME	0.056	0.038	1.465	0.143	−0.027	0.134
**Indirect effects**						
EXT → PA → PRS	0.016	0.007	2.202	0.028	0.002	0.034
EXT → PA → COR	0.012	0.010	1.196	0.232	−0.008	0.034
EXT → PA → SOS	0.038	0.010	3.806	<0.001	0.021	0.064
EXT → PA → EME	−0.019	0.007	−2.600	0.009	−0.037	−0.005
AGR → PA → PRS	0.031	0.009	3.581	<0.001	0.014	0.056
AGR → PA → COR	0.018	0.008	2.211	0.027	0.002	0.038
AGR → PA → SOS	0.013	0.011	1.198	0.231	−0.009	0.037
AGR → PA → EME	0.043	0.011	3.846	<0.001	0.024	0.071
CON → PA → PRS	−0.022	0.008	−2.609	0.009	−0.041	−0.006
CON → PA → COR	0.035	0.010	3.613	<0.001	0.016	0.061
CON → PA → SOS	0.015	0.007	2.185	0.029	0.003	0.033
CON → PA → EME	0.011	0.009	1.194	0.233	−0.008	0.032
NEU → PA → PRS	0.037	0.010	3.722	<0.001	0.021	0.062
NEU → PA → COR	−0.018	0.007	−2.572	0.010	−0.037	−0.005
NEU → PA → SOS	0.030	0.008	3.510	<0.001	0.014	0.054
NEU → PA → EME	0.015	0.007	2.162	0.031	0.002	0.030
OPE → PA → PRS	0.011	0.009	1.190	0.234	−0.008	0.030
OPE → PA → COR	0.035	0.010	3.618	<0.001	0.018	0.059
OPE → PA → SOS	−0.017	0.007	−2.538	0.011	−0.034	−0.004
OPE → PA → EME	0.028	0.008	3.422	<0.001	0.012	0.049

*EXT, extraversion; AGR, agreeableness; CON, conscientiousness; NEU, neuroticism; OPE, openness to experience; PRS, problem-solving; COR, cognitive restructuring; SOS, social support; EME, emotion expression; PA, positive affect*.

*Delta method standard error, bias-corrected percentile bootstrap confidence interval*.

As indirect effects, positive affect mediated in the relationship of extraversion with problem-solving, social support, and emotion expression. Positive affect also mediated in the relationships of the agreeableness factor with problem-solving, cognitive restructuring, and emotion expression.

Furthermore, positive affect was a mediator in the relationship of conscientiousness with the problem-solving, cognitive restructuring, and social support coping strategies. Finally, positive affect was observed to mediate in the relationships between openness to experience and cognitive restructuring, social support, and emotion expression.

Finally, the proportion of variance explained for each of the endogenous variables in Mediation Model 1 is the following: *R*^2^ = 0.237 for problem-solving, *R*^2^ = 0.116 for cognitive restructuring, *R*^2^ = 0.157 for social support, *R*^2^ = 0.096 for emotion expression, and *R*^2^ = 0.070 for the positive affect mediator.

Table 4 shows that the extraversion factor exerted a direct negative effect on the self-criticism and social withdrawal coping strategies. The agreeableness factor had a direct positive effect on wishful thinking and a negative effect on social withdrawal. The conscientiousness factor had a direct negative effect on the problem avoidance, social withdrawal, and self-criticism strategies, while the neuroticism factor exerted a direct positive effect on wishful thinking, social withdrawal, and self-criticism strategies.

**Table 4:** Direct and indirect effects (Model 2).

	**Estimated**	**Standard error**	***z*-value**	***p***	**95% CI**	
					**Below**	**Above**
**Direct effects**						
EXT → PRA	−0.013	0.036	−0.374	0.709	−0.091	0.057
AGR → PRA	−0.061	0.050	−1.219	0.223	−0.168	0.045
CON → PRA	−0.089	0.045	−1.967	0.049	−0.192	0.008
NEU → PRA	−0.062	0.036	−1.691	0.091	−0.137	0.009
OPE → PRA	0.028	0.039	0.711	0.477	−0.056	0.111
EXT → DES	−0.030	0.035	−0.858	0.391	−0.104	0.048
AGR → DES	0.209	0.049	4.254	<0.001	0.105	0.286
CON → DES	0.030	0.045	0.672	0.502	−0.058	0.124
NEU → DES	0.231	0.036	6.430	<0.001	0.159	0.310
OPE → DES	0.023	0.039	0.606	0.544	−0.057	0.096
EXT → SOW	−0.233	0.033	−6.953	<0.001	−0.306	−0.168
AGR → SOW	−0.179	0.046	−3.850	<0.001	−0.283	−0.084
CON → SOW	−0.141	0.042	−3.337	<0.001	−0.239	−0.053
NEU → SOW	0.102	0.034	2.993	0.003	0.029	0.168
OPE → SOW	−0.013	0.036	−0.353	0.724	−0.090	0.060
EXT → SEC	−0.117	0.033	−3.484	<0.001	−0.188	−0.037
AGR → SEC	0.017	0.046	0.361	0.718	−0.080	0.102
CON → SEC	−0.166	0.042	−3.941	<0.001	−0.258	−0.085
NEU → SEC	0.210	0.034	6.174	<0.001	0.145	0.291
OPE → SEC	−0.043	0.037	−1.176	0.239	−0.122	0.033
**Indirect effects**						
EXT → NA → PRA	−7.537e−4	0.006	−0.131	0.896	−0.014	0.010
EXT → NA → DES	−0.016	0.008	−1.934	0.053	−0.037	9.077e−5
EXT → NA → SOW	−0.017	0.008	−2.183	0.029	−0.038	−0.003
EXT → NA → SEC	0.046	0.010	4.664	<0.001	0.030	0.071
AGR → NA → PRA	2.623e−4	0.006	0.042	0.967	−0.013	0.013
AGR → NA → DES	−3.589e−4	0.003	−0.131	0.896	−0.008	0.006
AGR → NA → SOW	−0.008	0.005	−1.647	0.099	−0.020	−3.567e−4
AGR → NA → SEC	−0.008	0.005	−1.794	0.073	−0.021	−0.001
CON → NA → PRA	0.022	0.008	2.608	0.009	0.007	0.039
CON → NA → DES	1.249e−4	0.003	0.042	0.967	−0.006	0.008
CON → NA → SOW	−8.896e−4	0.007	−0.131	0.896	−0.016	0.012
CON → NA → SEC	−0.019	0.010	−1.977	0.048	−0.042	−1.782e−4
NEU → NA → PRA	−0.020	0.009	−2.245	0.025	−0.042	−0.003
NEU → NA → DES	0.054	0.010	5.379	<0.001	0.034	0.081
NEU → NA → SOW	3.096e−4	0.007	0.042	0.967	−0.016	0.015
NEU → NA → SEC	−0.001	0.008	−0.131	0.896	−0.018	0.014
OPE → NA → PRA	−0.022	0.011	−1.995	0.046	−0.045	9.475e−4
OPE → NA → DES	−0.023	0.010	−2.273	0.023	−0.048	−0.003
OPE → NA → SOW	0.063	0.011	5.795	<0.001	0.043	0.089
OPE → NA → SEC	3.579e−4	0.009	0.042	0.967	−0.018	0.018

*EXT, extraversion; AGR, agreeableness; CON, conscientiousness; NEU, neuroticism; OPE, openness to experience; PRA, problem avoidance; DES, desiderative thinking; SOW, social withdrawal; SEC, self-criticism; NA, negative affect*.

*Delta method standard error, bias-corrected percentile bootstrap confidence interval*.

As to the indirect effects, negative affect was observed to exert a mediating role in the relationship of extraversion with social withdrawal and self-criticism. Negative affect further mediated in the relationship of the conscientiousness factor with the problem avoidance and self-criticism coping strategies. For neuroticism, negative affect intervened as a mediator in its relationships with the problem avoidance and wishful thinking strategies. Finally, the mediator effect of negative affect was present in the relationships between the openness and problem avoidance, wishful thinking, and social withdrawal coping strategies.

Finally, the proportion of variance explained for each of the endogenous variables in Mediation Model 1 was the following: *R*^2^ = 0.033 for problem avoidance, *R*^2^ = 0.056 for wishful thinking, *R*^2^ = 0.156 for social withdrawal, *R*^2^ = 0.155 for self-criticism, and *R*^2^ = 0.075 for the negative affect mediator.

## Discussion

The objective of this study was to analyze the relationship of the coping strategies with affect and individual personality. In the first place, and in agreement with the literature (Hall et al., 2016; Hengartner et al., 2017), positive relationships were found between positive affect and all the Big Five Model traits except neuroticism, with which it shows a negative association, while the relationship of negative affect with the personality factors was the contrary.

In addition, concerning the relationships between affect and the coping strategies, a positive association was found between positive affect and adequate problem management or emotion, while negative affect showed a positive relationship mostly with strategies considered inadequate. Thus, while positive affect was especially related to active effort for compensating a stressful situation, negative affect would be linked to maladaptive coping with stress, showing a passive attitude by avoiding the problem. Previous studies have shown the negative role in harmful interpretation of the stressful situation, which could lead to overestimation of risk, and therefore avoiding confrontation with it or avoiding the distress associated with it (Dunkley et al., 2017). On the contrary, a positive affective state in stressful events means that one is active, lively, and alert, and so feels more confident of the ability of an individual to deal with the problem successfully, mainly by choosing active strategies (Cano-García et al., 2007).

The relationship between the Big Five factors and coping strategies was also confirmed, finding that the most prevalent personality traits considered more adaptive (extraversion, agreeableness, conscientiousness, and openness to experience) showed positive relationships with the more well-adjusted coping strategies and negative relationships with the negative coping strategies. Meanwhile, the neuroticism factor showed the opposite relationship with coping strategies, such that it associated positively with strategies typical of a negative approach and negatively with more adequate ways of managing the stressful event (Cano-García et al., 2007).

Then, the two mediation models were estimated. In the first model, it was observed that positive affect acted as a mediator in the relationship between neuroticism and all the positive strategies. Although this result may at first seem surprising, according to previous literature, this trait is especially linked to affect and regulation strategies (Pavani et al., 2017). In particular, individuals with high levels of neuroticism may select adaptive coping strategies when the affective burden of the situation is positive (Augustine and Larsen, 2011). Thus, even though emotional instability typical of the neuroticism factor usually leads to negative evaluation of the situation, and therefore a greater tendency to select negative solution strategies (Fornés-Vives et al., 2019), a person with high neuroticism may prefer strategies that promote adequate management in the presence of an enthusiastic, alive affective state. Similarly, those with personality traits considered positive select proactive problem-solving strategies and relieve the emotional distress through positive affect which means dealing with the situation actively and optimistically (Korotkov, 2008; Straud et al., 2015; Pavani et al., 2017).

The second model shows how negative affect mediated the effect of extraversion on inadequate emotion-focused management, wishful thinking, conscientiousness, problem avoidance, and self-criticism strategies; the neuroticism factor and inadequate problem-focusing management strategies; and the openness to experience trait and problem avoidance strategies, social withdrawal, and wishful thinking. Thus, when the affective state is marked by negative feelings, such as fear or guilt, coping strategies considered maladaptive may end up being employed, regardless of personality traits. Therefore, acquisition and development of effective coping strategies to solve the frequent stressful situations in the healthcare job context (Huang et al., 2018) goes through training in adequate problem management strategies as well as intervention on the affect generated by such events. This means undertaking the regulation dynamics between employee coping, affect, and personality together (De la Barrera et al., 2019).

It is important to state some limitations. In the first place, the characteristics of the stressful situation were not taken into account. The CSI asks the subject in the instructions to think of a situation experienced in the last month and evaluate the coping strategies used for it. As the situation remembered may have an affective load and different intensity in each case, and therefore be related to the type of coping selected, in future studies, the nature of the stressful event selected should be taken into consideration. We should also mention the short length of the instrument used to assess personality. Even though it is so short, it can be beneficial because it is easy to apply, although it may also reduce the measurement efficacy. Variables such as age, years of experience, or the department where they were working were not taken into account either. In future studies, these factors should be controlled for, given their connection with coping strategies.

## Conclusions

Since the exposure of nurses to stress is so high, inadequate management can cause more vulnerability to the development of physical and psychological affects, while adaptive coping promotes wellbeing and effective solution. This study showed that personality can be closely linked to the way worker stress is handled. However, the affective state mediates in this relationship, such that when negative affect is experienced, stressful events are managed less adaptively, and when positive affect is present, it acts as a mediator between personality factors and positive coping strategies.

Therefore, this study shows the need to continue inquiry into factors involved in effective coping with stressful situations in the nursing work environment and variables related with their appearance. Their in-depth knowledge would make it possible to develop actions teaching adequate problem-solving strategies and training in the ability to assign a different emotional value to complicated situations.

## Data Availability Statement

The original contributions presented in the study are included in the article, further inquiries can be directed to the corresponding author.

## Ethics Statement

The studies involving human participants were reviewed and approved by the Bioethics Committee of the University of Almería (Ref: UALBIO2017/011). The patients/participants provided their written informed consent to participate in this study.

## Author Contributions

MCP-F, MMMJ, ÁMM, ABBM, and MMSM contributed to the conception and design of the review. JJGL and ABBM applied the search strategy. MCP-F, MMMJ, and ÁMM wrote this manuscript. MCP-F and JJGL edited this manuscript. All authors applied the selection criteria, completed the assessment of the risk of bias, analyzed the data and interpreted data, and read and agreed to the published version of the manuscript.

## Conflict of Interest

The authors declare that the research was conducted in the absence of any commercial or financial relationships that could be construed as a potential conflict of interest.

## Publisher's Note

All claims expressed in this article are solely those of the authors and do not necessarily represent those of their affiliated organizations, or those of the publisher, the editors and the reviewers. Any product that may be evaluated in this article, or claim that may be made by its manufacturer, is not guaranteed or endorsed by the publisher.
